# Fabrication, Characterization, and In Vitro Digestion Behavior of Bigel Loaded with Notoginsenoside Rb1

**DOI:** 10.3390/gels11080624

**Published:** 2025-08-09

**Authors:** Yang Luo, Gao Xiong, Xiao Gong, Chunlei Xu, Yingqiu Tian, Guanrong Li

**Affiliations:** 1 College of Notoginseng Medicine and Pharmacy, Wenshan University, Wenshan 663099, China; 2 Agricultural Products Processing Research Institute, Chinese Academy of Tropical Agricultural Sciences, Zhanjiang 524001, China; 3 College of Agronomy and Biotechnology, Southwest University, Chongqing 400715, China

**Keywords:** bigel system, oleo-hydrogel hybrid, notoginsenoside Rb1, encapsulation, characterization, in vitro digestion

## Abstract

Notoginsenoside Rb1 (Rb1), a bioactive saponin from *Panax notoginseng,* exerts cardio-cerebrovascular protective, anti-inflammatory, antioxidant, and glucose homeostasis-regulating effects. However, its oral bioavailability is limited by gastric degradation and poor intestinal permeability. This study presents a food-grade bigel system for encapsulating Rb1 to enhance its stability and controlled-release performance. Oleogels were structured using monoglycerides (8%, *w*/*w*) in soybean oil. Rb1-loaded binary hydrogels (gellan gum/xanthan gum, 12:1 *w*/*w*) were emulsified in 10% Tween-80 (*w*/*w*). Bigels were formulated at varying hydrogel-to-oleogel ratios, and a ratio of 4:6 was identified as optimal. Stress-sweep rheological analysis revealed a dense gel structure with a peak storage modulus (G′) of 290.64 Pa—the highest among all tested ratios—indicating superior structural integrity. Confocal microscopy confirmed homogeneous encapsulation of Rb1 within the continuous hydrogel phase, effectively preventing payload leakage. Differential scanning calorimetry (DSC) analysis detected a distinct endothermic transition at 55 °C (ΔH = 6.25 J/g), signifying energy absorption that enables thermal buffering during food processing. The system achieved an encapsulation efficiency of 99.91% and retains both water and oil retention. Effective acid protection and colon-targeted delivery were observed in the digestion test. Effective acid protection and colon-targeted delivery were observed in the digestion test. Less than 5% of Rb1 was released in the gastric phase, and over 90% sustained intestinal release occurred at 4 h. The optimized bigel effectively protected Rb1 from gastric degradation and enabled sustained intestinal release. Its food-grade composition, thermal stability, and tunable rheology offer significant potential for use in functional foods and nutraceuticals.

## 1. Introduction

*Panax notoginseng*, a traditional medicinal herb rich in dammarane-type saponins, contains abundant amounts of the pharmacologically notoginsenoside Rb1 (Rb1) [1]. Rb1 presents a broad spectrum of bioactivities, including anti-inflammatory, antioxidant, anti-fatigue, and antitumor effects [2,3]. It is particularly notable for its cardioprotective properties, such as inducing vasodilation, reducing myocardial oxygen consumption, alleviating angina, enhancing tolerance to cerebral ischemia, and improving immune function. Rb1 also exhibits glucose-regulating effects beneficial for diabetes management [4,5,6]. However, its efficacy following oral administration is significantly limited by its instability upon exposure to high temperatures, light, oxygen, and gastric acid, coupled with poor intestinal permeability, which results in low oral bioavailability [7,8,9,10].

Bigels, a class of biphasic systems composed of hydrogels and oleogels, have emerged as versatile carriers of bioactive compounds with both hydrophilic and lipophilic characteristics. They combine the desirable properties of both gel types and offer enhanced mechanical strength, phase compatibility, and controlled release capabilities [11,12]. Bigels have been increasingly studied for food and pharmaceutical applications owing to their ability to stabilize and deliver sensitive functional ingredients.

Recent research has demonstrated that the physicochemical properties and structural behavior of bigels are critically influenced by their composition and hydrogel-to-oleogel ratio. For instance, Yang et al. (2022) reported that sodium alginate/glycerol monostearate (GMS) bigels showed near-complete oil retention at low oleogel contents, with freeze–thaw stability improving as GMS concentrations increased [13]. Furthermore, the continuous-phase-type oil-in-water (O/W), water-in-oil (W/O), or bicontinuous phase can be precisely tailored by modulating this ratio. Studies have established that phase inversion occurs progressively with increasing oleogel content, significantly impacting droplet size, phase stability, and emulsion microstructure [14,15]; these collective findings confirm that hydrogel-to-oleogel ratios critically determine continuous phase behavior, directly influencing physical stability and functional properties in food applications [16,17,18].

Current gel systems have primarily focused on delivering fat-soluble functional ingredients, while water-soluble functional ingredients are more susceptible to degradation during processing and storage. Aqueous solubility of Rb1 makes it highly susceptible to destruction in the digestive tract. The double-layer embedded gel system showed advantages in terms of structural protection and delivery enhancement and offers a novel solution for stabilizing Rb1. In this work, a biphasic gel system for Rb1 was obtained, and the hydrogel-to-oleogel ratio was optimized. This system aims to overcome gastric degradation and achieve controlled sustained release in the intestine. A bicontinuous structure with an optimized hydrogel–oleogel ratio revealed excellent stability and functionality and enhanced the delivery efficiency of Rb1. This work developed a food-grade biphasic gel system for Rb1 encapsulation, which strengthened Rb1’s protective capacity under simulated gastrointestinal conditions and realized its controlled intestinal release, suggesting a promising potential in functional foods and nutraceuticals.

## 2. Results and Discussion

### 2.1. Texture Properties

Mechanical properties of bigels critically determine their suitability for food and pharmaceutical applications. Texture profile analysis (Table 1) revealed a decrease in hardness for both the oleogel and hydrogel phases upon mixing. Hardness represents the structural strength of the gel network and directly correlates with deformation resistance, so higher oleogel fractions usually yield firmer textures. The pure oleogel (1:0) exhibited the highest hardness (928.62 ± 0.64 gf). When the hydrogel proportion increased from 1:9 to 5:5, its hardness significantly increased from 33.25 to 79.85 gf due to aqueous phase disruption of the oleogel’s continuous phase. The pure hydrogel (0:1) demonstrated the lowest hardness (31.74 ± 0.96 gf) and showed the characteristic of soft gel structures.

Cohesiveness reflects fracture resistance, and higher cohesiveness reduces structural disintegration during mastication. The pure oleogel (1:0) shows minimal cohesiveness (0.27 ± 0.01), indicating brittle behavior. Mixed gels (1:9 to 5:5) exhibit enhanced cohesiveness (from 0.79 to 0.81), which is attributable to stable oil–water interfacial double-network formation. The pure hydrogel (0:1) displays intermediate cohesiveness (0.72 ± 0.01), which is consistent with partial recoverability of hydrated networks.

Springiness describes post-deformation recovery speed/extent, representing shape-restoration capacity. The pure oleogel (1:0) exhibits negligible springiness (0.26 ± 0.00 mm) with plastic dominance. Increasing the hydrogel content progressively elevates springiness (from 0.78 mm at the ratio of 1:9 to 0.83 mm at the ratio of 5:5) due to the aqueous phase imparting entropic elasticity through polymer-chain retraction. While the pure hydrogel (0:1) shows moderate springiness (0.76 ± 0.00 mm), the 5:5 blend achieves superior recovery, demonstrating oil–water synergy.

Gumminess characterizes the energy required for semi-solid disintegration prior to swallowing. The pure oleogel (1:0) demands exceptionally high energy (247.03 ± 0.01 gf), reflecting slow oral meltdown akin to butter. Mixed gels require substantially lower gumminess (from 26.44 gf at the ratio of 1:9 to 63.95 gf at the ratio of 5:5), facilitating rapid oral dispersion. The pure hydrogel (0:1) exhibits minimal gumminess (22.73 ± 0.01 gf), enabling immediate breakdown, which reflected the viscous resistance of semi-solid gels, with higher oleogel content increasing oral processing demands.

Chewiness is related to the energy required to achieve swallow-ready consistency. The pure oleogel (1:0) shows moderate chewiness (64.10 ± 0.04 gf) due to high hardness offset by low elasticity. The 5:5 blend achieves peak chewiness (53.28 ± 0.10 gf) through synergistic hardness–cohesiveness–springiness interactions. High-water-content gels (e.g., 0:1) display minimal chewiness (17.27 ± 0.01 gf), requiring negligible mastication.

The 5:5 gel excels in cohesiveness and springiness, making it ideal for elastic, chewable products. Conversely, the 4:6 formulation demonstrates superior balance between moderate hardness (67.96 gf) and reduced chewiness (40.44 gf), rendering it optimal for soft, easily processed foods. Generally, as the hydrogel content increased, the overall hardness of the bigel decreased. This trend aligns with the findings reported for similar bigel systems [19]. The reduction in hardness is attributed to dilution effects from the aqueous hydrogel phase and the weakening of the compact matrix of the oleogel. Significant differences (*p* < 0.05) were observed among various hydrogel-to-oleogel ratios. This reduction is attributed to dilution effects from the aqueous phase and the disruption of the oleogel’s compact matrix [20]. The oleogel-dominant formulation (1:0) exhibited the highest hardness, chewiness, and gumminess, reflecting its uninterrupted crystalline network and strong van der Waals interactions. Low hydrogel fractions (<40%) preserve oleogel integrity but limit water-soluble compound loading. Conversely, high hydrogel fractions (>60%) weaken the matrix via competitive hydrogen bonding, which displaces oleogelator self-assembly. The 4:6 formulation demonstrated the most balanced textural properties, consistent with its stable microstructure.

### 2.2. Microstructure Observation of Bigels

**Figure 1:**
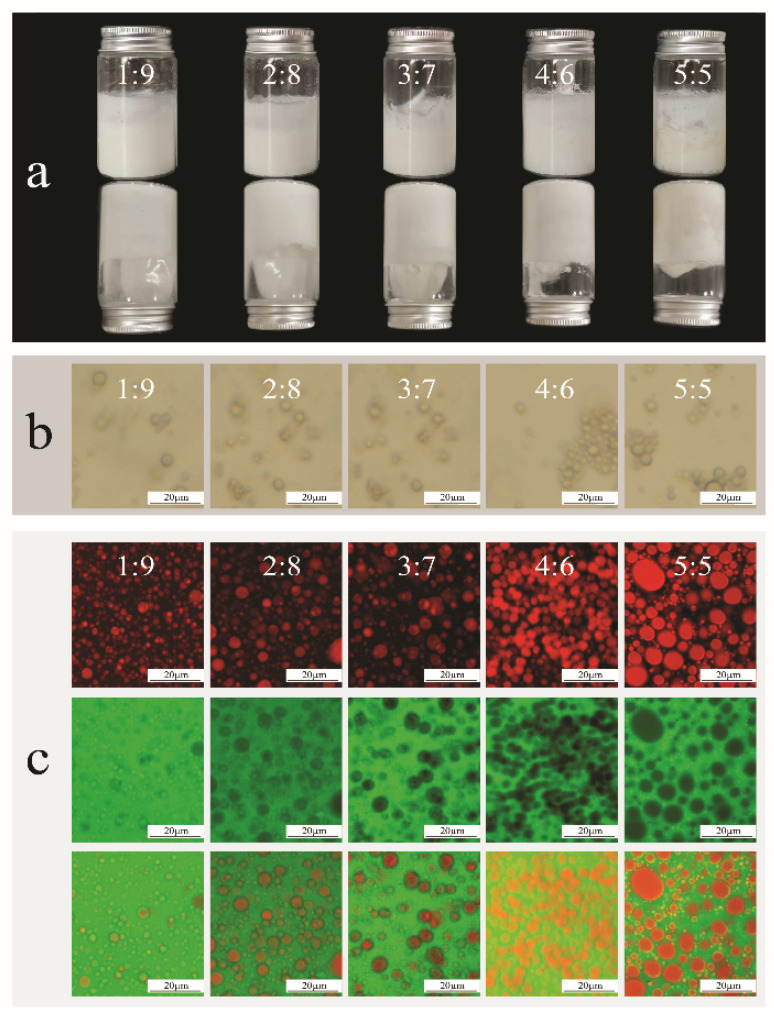
Microstructure of bigels at different ratios: (**a**) visual appearance; (**b**) bright-field microscopy; (**c**) confocal laser scanning microscopy.

Gellan gum, a linear anionic polysaccharide, functions as a gel skeleton. In contrast, xanthan gum is a rigid, rod-like polyanion that exhibits a high water-holding capacity and pseudoplasticity, rendering it effective as a gel network enhancer. Gellan gum forms double helices that crosslink into dense networks, thereby enhancing the hydrogel’s strength and elasticity [21,22]. Variations in bigel proportions revealed that adding Rb1 did not alter the hydrogel states. Macroscopically, high-speed shearing produced milky-white bigels (Figure 1a). At low oleogel-to-hydrogel ratios (1:9–4:6), the bigels exhibited a uniform texture, layer resistance, and excellent stability. Conversely, at a 5:5 ratio, the gels developed a light-yellow coloration, an uneven texture, and flocculent stratification, indicating structural instability. Further analysis showed higher fluidity in bigels with ratios of 1:9 to 3:7, suggesting loose internal structures with homogeneously dispersed oil droplets lacking distinct continuous phases. At a 5:5 ratio, oil droplet aggregation increased substantially, reducing fluidity and causing surface roughness and stratification, likely because of the interfacial tension imbalance from the elevated oleogel content. The bigel systems composed of varying proportions of oleogels and hydrogels were examined at the same magnification using a bright-field microscope (Figure 1b). A significant difference in droplet size was observed. When the ratio was 4:6 or 5:5, the droplet distribution was uniform. Specifically, at a ratio of 4:6, the droplets were small and uniform, suggesting a stable structure. However, at the 5:5 ratio, the oil droplets were larger and irregularly shaped, potentially linked to the dynamic equilibrium of oleogel–hydrogel interactions. This could explain the difficulty in forming regular shapes when the oil droplets were dispersed within the hydrogel phase, aligning with the stability test results. Furthermore, when the oleogel-to-hydrogel ratio was 1:9, 2:8, or 3:7, small regular spherical oil droplets were observed. Notably, a typical oil-in-water (O/W) structure was observed at a 1:9 ratio, where the hydrogel served as a continuous phase, restricting and constraining the oil droplets to a spherical shape with low surface energy. As the proportion of the oleogel increased, the system progressively transitioned to a bicontinuous phase structure, likely because a new equilibrium state was established between the oil and water phases.

Laser scanning confocal microscopy (LSCM, Leica, Wetzlar, Germany) revealed the microstructure of the oleogel–hydrogel blends at various ratios (Figure 1c). With the oleogel and hydrogel pseudo-colored red and green, respectively, the 5:5 blend exhibited large, irregular oil droplets. This morphology likely reflects the dynamic oleogel–hydrogel equilibrium, where interfacial tension and intermolecular forces hinder regular spheroid formation in hydrogel-dispersed systems. Conversely, the ratios of 1:9–4:6 showed smaller spherical droplets. The 1:9 ratio formed distinct oil-in-water (O/W) structures, confirming that the hydrogel‘s continuous phase confined the droplets to low-surface-energy spheres. The progressive increase in oleogel content induced bicontinuous phase transitions, demonstrating oil–water re-equilibration [23].

### 2.3. Bigel Stability Analysis of Bigels

**Figure 2:**
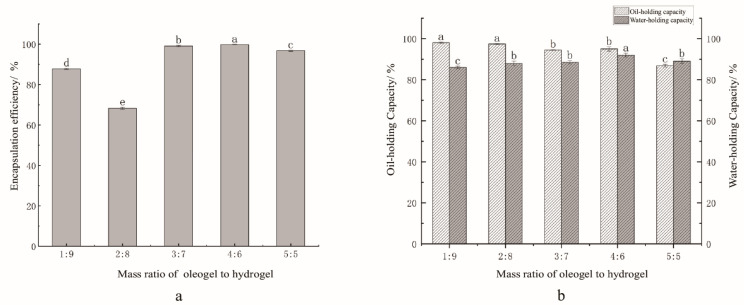
Bigel stability analysis. (**a**) Rb1 encapsulation efficiency; (**b**) freeze–thaw performance. Note: Different letters denote significant differences (*p* < 0.05).

Centrifugal and freeze–thaw stability assessments confirmed that Rb1 stability correlates directly with bigel integrity. The oil-to-water ratio critically modulates the biphasic networks, synchronously governing encapsulation efficiency (EE) and phase retention (Figure 2). At the optimal 4:6 ratio, a dense uniform network achieved the peak Rb1 EE (99.91%, vs. 62.28% for 2:8) (Figure 2a) and the maximum water-holding capacity (WHC, 91.98%, vs. + 6.98% for 1:9); all bigels have superior oil-holding capacity (OHC > 86%) (Figure 2b). This optimization stems from the dual functionality of the oleogel as a flexible filler, cooperating with the hydrogel to inhibit oil migration (interfacial stabilization) while alleviating network contraction (stress buffering). Deviations from the 4:6 ratio induced syneresis through hydrophilic–lipophilic balance disruption or network over-densification, causing EE decline (EE, 99.91%, vs. + 96.78% for 5:5), WHC reduction, and OHC deterioration. The 4:6 system achieved multifunctional synergy through pore confinement (enhanced EE), interfacial stabilization (oil retention), flexible buffering (WHC maintenance), and hydrophobic barriers (freeze–thaw resistance), thus providing a structural blueprint for functional bigels in food systems.

Stability tests, including centrifugation and freeze–thaw cycles, demonstrated that the 4:6 formulation had superior encapsulation efficiency (99.91%). Nano/microscale domains physically entrap Rb1 molecules’water-holding capacity (91.98%) and oil-holding capacity (95.00%) compared to the other ratios (Figure 2b). These results suggest that this composition achieved optimal synergy between the hydrophilic and hydrophobic phases. Deviations from the 4:6 ratio resulted in decreased stability owing to interfacial or network–structure imbalances. These findings confirm the multifunctional protective mechanisms of bigels, such as interfacial stabilization, structural confinement, and mechanical resilience, which are vital for maintaining their bioactive integrity [24]. The bigel system is an effective carrier for Rb1. The retention of Rb1 is related to phase continuity and integrity, and synchronized interfacial stabilization and mechanical buffering abilities will limit the applications in the functional food and pharmaceutical industries.

### 2.4. Fourier Transform Infrared (FTIR) Spectroscopy Analysis of Bigels

**Figure 3:**
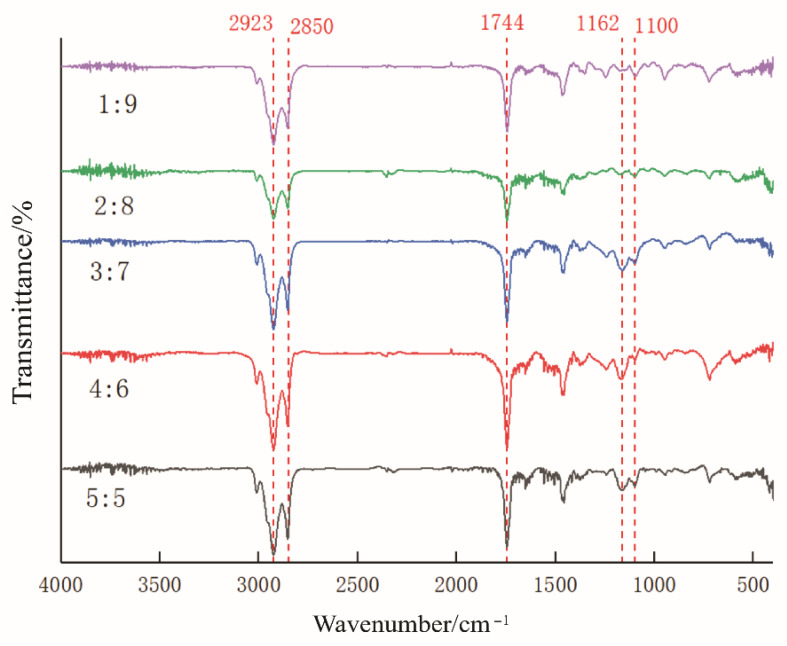
Fourier transform infra-red (FTIR) spectra of bigel systems.

Fourier transform infrared spectroscopy elucidated oleogel–hydrogel interactions in bigels, revealing distinct C-H stretching vibrations at 2923 cm^−1^ and 2850 cm^−1^ (Figure 3), characteristic of the oleogel -CH_2_ groups, while the C=O stretching peak at 1744 cm^−1^ confirmed abundant ester bonds in soybean oil and monoglycerides. As the hydrogel content increased, the C=O signal decreased, suggesting physical encapsulation or dilution [25]. Hydrogel polymers encapsulated/isolated the oil- phase of C=O groups during mixing, thus hindering infrared interactions. Concurrently, emerging C-O stretching peaks at 1162 cm^−1^ and 1100 cm^−1^ indicated that hydrogel incorporation- induced system reorganization [26], with these functional group distributions and interactions aligning with the findings by Zhou et al. (2025).

Spectral transitions (C=O peak attenuation, emergent C-O peaks, and persistent C-H vibrations) align with functional performance in food systems. The C=O shielding effect enables gastric-targeted release in acidic products. C-O network reinforcement supports the additive manufacturing of complex-textured foods (e.g., structural fidelity in 3D-printed plant-based meats), and C-H bond stability ensures thermal resilience in retorted snacks. This molecular-level understanding facilitates the rational design of bigels, paving the way for synergistic advancements in precision nutrition delivery and advanced food structuring technologies.

### 2.5. Differential Scanning Calorimetry (DSC) Analysis of Bigels

**Figure 4:**
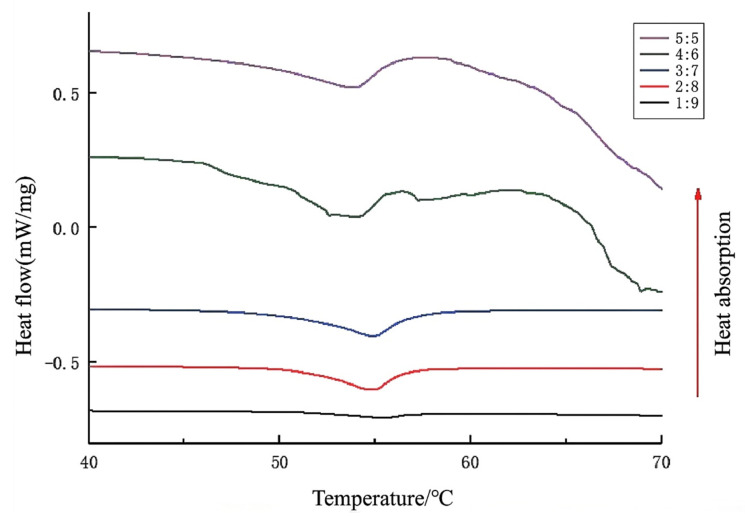
Differential scanning calorimetry (DSC) thermograms of bigels with varying oleogel/hydrogel ratios.

DSC thermograms of Rb1-loaded bigels exhibited an obvious endothermic transition between 48 and 62 °C (Figure 4). The onset temperature (48.2 °C) marks the initial disassembly of monoglyceride crystal networks, while the peak temperature (55.0 °C) corresponds to maximal lattice energy dissipation. The final temperature (61.5 °C) confirms full transition to a fluid state. The significant enthalpy change (ΔH = 6.25 J/g) reflects the energy required to disrupt oleogel crystallinity and can affect the thermal stability of the gel system of Rb1. Consequently, an increased proportion of these thermally active components in the system leads to a significant increase in the intensity of the endothermic peak [13].

The bigel system exhibits strong heat absorption near 55 °C, particularly at the ratio of 4:6. This property can enable its use as a thermal buffer or protective agent during food processing. Bigels can encapsulate thermally sensitive functional components (such as probiotics, vitamins, antioxidants, and flavor compounds). By absorbing thermal energy, they reduce the core temperature and effectively preserve the activity and flavor integrity of these components. The significant endothermic peak observed near 55 °C indicates a phase transition point at which the structure of the system undergoes key changes that impact its rheology and permeability. This behavior provides a basis for designing temperature-responsive controlled-release food carriers. Such carriers can maintain structural stability at oral temperature (about 37 °C), encapsulating flavors or nutrients, while undergoing structural changes upon reaching approximately 55 °C (e.g., during cooking or specific processing stages) or in targeted physiological environments, thus enabling triggered release.

### 2.6. Determination of Rheological Properties of Bigels

**Figure 5:**
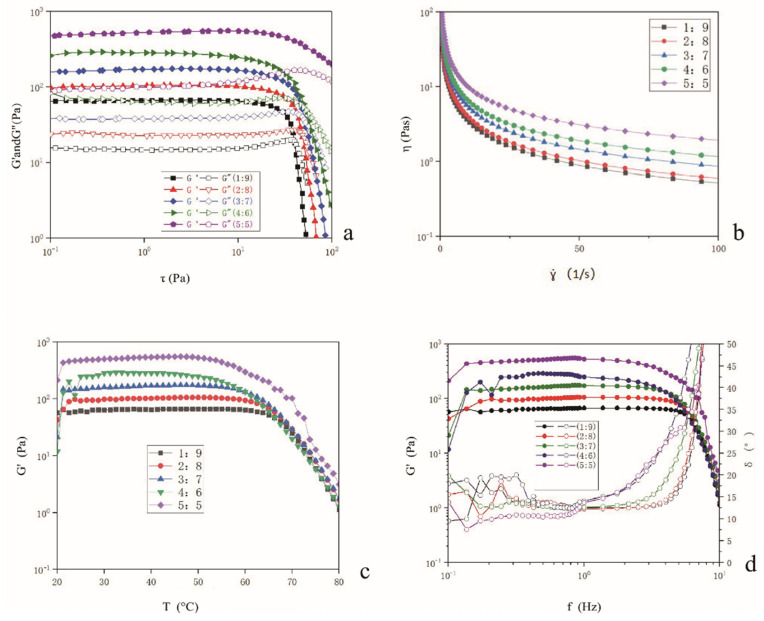
Rheological characteristics of bigels: (**a**) stress scan; (**b**) shear rate dependence; (**c**) temperature sweep; (**d**) frequency scan.

Five different bigel system proportions (1:9, 2:8, 3:7, 4:6, and 5:5) exhibited linear viscoelasticity within the stress range of 0.1–10 Pa (Figure 5a). By comparing the relationship between the storage modulus (G′) and the loss modulus (G″), the stress scanning curves of the five different proportions of bigels showed that G′ > G″, indicating gel properties [27]. The higher storage modulus G′ reflects a more compact structure of the sample. When comparing the storage modulus G′ of the five different proportions of bigels, the stable bigel system with an oleogel-to-hydrogel ratio of 4:6 showed the highest G′ value of 290.64 Pa, whereas the lowest G′ value was 100.73 Pa, suggesting that the network structure was the most compact and stable.

The bigels exhibited pseudoplastic behavior at various ratios. As the shear rate increased, the viscosity of the bigels decreased (Figure 5b), characteristic of a typical non-Newtonian fluid. This is primarily attributed to three factors. First, at the molecular level, in a static state, molecular chains are entangled and cross-linked to form a three-dimensional network structure with high viscosity. Upon exposure to a shear force, the molecular chains align in the direction of shear, the winding structure is disrupted, and interactions are weakened, leading to a reduction in viscosity. Second, from the perspective of the internal structure of the fluid, micelles, microemulsions, and other microstructures remain stable and maintain high viscosity at low shear rates. However, high shear rates can cause these structures to deform, rupture, or rearrange, thereby altering the fluid flow properties. Third, considering the solvation effect, the solvation layer formed by the polymer and the solvent in the bigels is compromised during shearing. The higher the shear rate, the more severe the damage, which facilitates free movement of the polymer within the solvent, resulting in decreased fluid viscosity. At low shear rates, viscosity represents the storage capacity and stability of the fluid. As the proportion of oleogels to hydrogels increased, viscosity also increased accordingly. With an increase in shear rate, the properties of the five types of bigels were similarly affected. When the ratio of oil to hydrogels was 4:6, the effect of the shear force was minimal, which may be attributed to the denser network structure formed by the oleogel, effectively resisting the shear force and diminishing the sensitivity of the viscosity to the shear rate [28].

The effect of temperature on the storage modulus (G′) of the bigels (Figure 5c) indicates that the G′ value gradually decreases as temperature increases. This may be due to the fact that with rising temperatures, the crystallization of the outer oleogel begins to melt, the crystal network structure starts to deteriorate, and the three-dimensional network structure ‘collapses’. Bigels in different proportions exhibited significant variations in their melting characteristics. Specifically, bigels with a 4:6 ratio began to melt first, followed by those with ratios of 5:5, 3:7, 2:8, and finally 1:9. There could be two reasons for this. First, the degree of interaction between the oleogel and the hydrogel in the bigels and the crystallization melting temperature of the oleogel within the bigels are related. Upon reaching the critical temperature of 45 °C, the storage modulus of the five bigels decreases rapidly, suggesting that the oleogel ruptures and stability begins to diminish. The most rapid decline in G′ occurs within the temperature range of 65–80 °C, which may be associated with oleogel rupture and further destruction of the network structure.

The frequencies and rheological properties of five different proportions of the oil and hydrogel systems were examined. In the low-frequency range of 0.1 to 6 Hz, each system exhibited a typical gel behavior, characterized by G′ > G″ (Figure 5d), indicating that elasticity was maintained through an internal three-dimensional network structure. When the frequency surpassed 6 Hz, a gel–solution transition occurred (phase transition angle: δ > 45°), and the phase transition angle exceeding 45° signified that the bigels shifted from elasticity to viscosity dominance. Notably, bigels with a 4:6 mass ratio were the first to undergo a phase transition, which may be attributed to their unique phase structure and component interactions. Following the time–temperature equivalence principle, high-frequency equivalent heating accelerates the phase transition of the heat-sensitive component of bigels. Concurrently, a high frequency reduces the viscosity of the system and enhances its fluidity. These factors contribute to the phase transition angle exceeding a critical value. The variance in the frequency response among systems with different mass ratios fundamentally stems from differences in the internal phase structure, intermolecular forces, and component synergistic effects.

### 2.7. Determination of the Rb1 Release Rate During Digestion

**Figure 6:**
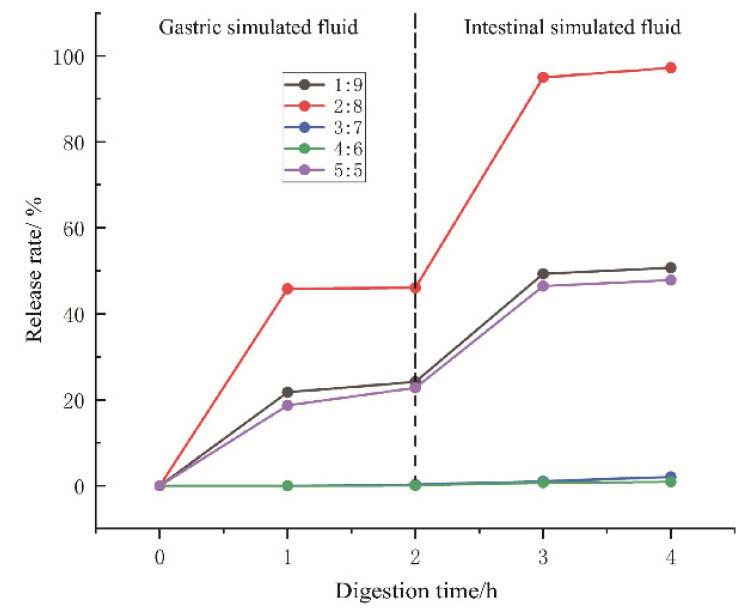
Release profile of Rb1 during simulated digestion.

The capacity of bigel systems with varying proportions for Rb1 loading and release behavior was investigated in a simulated digestive environment. Experimental results in gastric and intestinal simulated solutions (Figure 6) indicated that a 2:8 mass ratio of oleogels to hydrogels resulted in a relatively high release rate of Rb1. This can be attributed to the surface being predominantly composed of hydrogels. In the simulated gastric solution, the hydrophilicity of the hydrogel causes it to swell and rupture upon contact with gastric fluid, thereby releasing the encapsulated Rb1. The surface hydrophilicity of the oil-in-water bigels further enhances their swelling capacity and promotes the release of active ingredients [29]. In simulated intestinal fluid, the release rate of notoginsenoside Rb1 was significantly increased, which may be related to the degradation caused by lipases and proteases in the intestinal fluid. Intestinal fluid further weakens the hydrogel structure, resulting in its complete destruction. Simultaneously, oil decomposition deforms the network structure of the bigels, facilitating the release of Rb1 [30]. The high permeability of the intestinal fluid may also accelerate the diffusion of active ingredients.

Throughout the digestive system, Rb1 showed a trend of sustained release, suggesting that the bigel system possessed favorable sustained-release properties. Overall, the bigel system with an oleogel-to-hydrogel mass ratio of 4:6 demonstrated a relatively favorable sustained release effect on Rb1, aligning with the characteristics of controlled release [31,32]; over 90% sustained intestinal release occurred at 4 h.

At a ratio of 2:8 (high release), the surface predominantly consists of the hydrogel (water-continuous phase), enabling rapid swelling in gastric fluid and efficient Rb1 release. Hydrogel continuity facilitates direct contact with digestive fluids, accelerating rupture and release. At a 1:9 ratio (lower release), an excessive hydrogel fraction triggers phase inversion (oil-in-water to water-in-oil transition). The hydrogel matrix becomes highly viscous/rigid (higher G′ at low oleogel content), slowing diffusion and delaying Rb1 release. At a 3:7 ratio (moderate), G′ values were between those at the 2:8 and 4:6 ratios (Figure 5a). When the structure is less compact than the 4:6 ratio but more structured than the 1:9/2:8, the oleogel fraction is sufficient to form semi-continuous domains, partially shielding Rb1. Release is slower than the 2:8 ratio due to reduced hydrogel surface exposure. It melts earlier than that of the 4:6 ratio (Figure 5c), indicating weaker thermal resilience, which moderately accelerates Rb1 release in warm digestive conditions. The 4:6 bigel system (sustained), with the highest storage modulus (G′ = 290.64 Pa), has a dense, cohesive network (Figure 5a). The oleogel forms a continuous phase intertwined with the hydrogel, physically encapsulating Rb1. A strong endothermic peak at 55 °C (Figure 4) confirms energy absorption by oleogel components, stabilizing the structure during thermal stress (e.g., digestion). The attenuated C=O peak (1744 cm^−1^) confirms oleogel encapsulation (Figure 3), reducing enzymatic/lipid degradation in intestines. In terms of controlled release, gradual Rb1 diffusion occurs as digestive enzymes slowly disrupt the oleogel–hydrogel interface.

The 4:6 bigel system, characterized by high mechanical strength and precise enzyme responsiveness, can thus serve as a critical material for developing high-end functional foods, enabling differential gastrointestinal release systems (e.g., exercise supplements) and functioning as an intelligent delivery carrier for active ingredients (probiotics and vitamins) to achieve targeted controlled release and enhanced bioavailability.

## 3. Conclusions

A food-grade bigel system was successfully developed using gellan and xanthan gums as a binary hydrogel matrix, monoglyceride-structured soybean oil as the oleogel phase, and Tween-80 as an emulsifier for encapsulating Rb1. Among the tested formulations, the 4:6 oleogel-to-hydrogel ratio showed the most compact and stable microstructure, demonstrating exceptional encapsulation efficiency, mechanical properties, and sustained-release performance under simulated gastrointestinal conditions. This controlled release profile was governed by hydrogel swelling with enzymatic hydrolysis (gastric phase) followed by network disruption (intestinal phase), while oleogel incorporation enhanced structural integrity, diffusion resistance, and thermal stability. Nevertheless, in vitro gastrointestinal models fail to fully replicate critical physiological aspects of the human GI tract, including mucosal dynamics and gut microbiota interactions; the release kinetics of Rb1 may differ in humans. Real practical applications often involve multicomponent systems such as sweeteners, dyes, or emulsifiers, which may alter performance. Consequently, in vivo animal studies are required to quantify the bioavailability enhancement of encapsulated Rb1 and other bioactives versus free compounds or conventional delivery systems. Collectively, the Rb1-loaded bigel system demonstrates significant potential as a delivery platform for enhanced bioavailability and functionality, supporting applications in functional foods and nutraceuticals.

## 4. Materials and Methods

### 4.1. Materials

Notoginsenoside Rb1 (≥80%) was purchased from Shaanxi Taike Biotechnology Co., Ltd. (Xi’an, China). Soybean oil was obtained from Jiusan Food Co., Ltd. (Harbin, China). Monoglycerides were obtained from Galix Additives Co., Ltd. (Shanghai, China). Gellan and xanthan gums were supplied by Wanbang Chemical Technology Co., Ltd. (Zhengzhou, China). Tween-80 (TW-80) was obtained from Aobin Biotechnology Co., Ltd. (Guangzhou, China). Nile red and fluorescein isothiocyanate isomer I (FITC) were obtained from McLean Biochemical Technology Co., Ltd. (Shanghai, China) and Dulai Biotechnology Co., Ltd. (Nanjing, China), respectively. Acetonitrile and methanol were purchased from Bolinda Technology Co., Ltd. (Shenzhen, China). Artificial simulated gastric fluid (pH 1.5, 10 g/L pepsin) and intestinal fluid (pH 6.8 ± 0.1, 10 g/L trypsin) were provided by Liquid Wide Detection Technology Co., Ltd. (Suzhou, China).

### 4.2. Preparation of Oleogels

Oleogels were prepared by dissolving 8% (*w*/*v*) the mass fraction of the monoglyceride in soybean oil under constant stirring at 70 °C; the solution was then cooled to room temperature to solidify [33].

### 4.3. Preparation of a Rb1-Loaded Hydrogel

Hydrogels were prepared by dissolving 3% of the gels (the ratio of gellan gum to xanthan gum is 12:1) in deionized water at 75 °C under continuous stirring; the solution was then cooled to room temperature to form a uniform hydrogel [34].

For Rb1 loading, Rb1 was dissolved using an ultrasonic processor (model SB-5200DT, Scientz, Ningbo, China) operating at a frequency of 40 kHz with an output power of 360 W in continuous mode for 5 min in deionized water at 1.0 g/L. Then the pH of the solution was adjusted to approximately 4.0 using 6 mol/L HCl. The Rb1 solution was mixed at a fixed mass ratio of 1:1 (*w*/*w*), followed by adding 10% (*w*/*w*) TW-80. The mixture was heated to 70 °C, and then allowed to stand for 30 min to remove air bubbles and solidify.

### 4.4. Preparation of Rb1 Bigels

Hydrogels containing Rb1 with oleogels were mixed at different mass ratios (1:9, 2:8, 3:7, 4:6, and 5:5) at 70 °C, and then immediately homogenized at 20,000 rpm for 5 min using a high-shear homogenizer (model FJ200-S, Lichen, Shenzhen, China) to obtain bigels loaded with Rb1.

### 4.5. Texture Analysis

Texture profile analysis (TPA) was performed using a TA texture analyzer (model, XTC-18, Baosheng, Shanghai, China) equipped with a P/45 conical probe, as described by Jin et al. (2021). The testing parameters were as follows: a probe speed of 1 mm/s, a penetration depth of 1 mm, a travel distance of 60 mm, and a trigger force of 5 g. All measurements were conducted in triplicate, and the mean values were calculated [35].

### 4.6. Microstructure Observation

The microscopic morphology was observed using a Leica microscope (Leica Mica, Germany) equipped with a 40 × objective lens, and bright-field micrographs were captured using this system. For fluorescence imaging, oleogel components were stained with Nile red (1 mg/mL in ethanol), and hydrogel components were stained with FITC (1 mg/mL in ethanol), followed by incubation in the dark (15 min, 25 °C). Confocal laser scanning microscopy (CLSM) was performed using a Leica system with a 63 × oil immersion objective. FITC was excited at 488 nm with emission assessed at 500–550 nm, and Nile Red was excited at 552 nm with emission at 570–620 nm [36].

### 4.7. Bigel Stability Analysis

The stability of bigels (double-gel system) was evaluated using freeze–thaw stability testing as described by Guo (2023), wherein the water-holding capacity (WHC) and oil-holding capacity (OHC) were calculated based on the mass of the separated upper oil and lower water phases after centrifugation [15]. Additionally, the embedding rate of Rb1 within the bigels was determined by centrifugation using the method described by Yan et al. (2019) and Liu et al. (2020) with slight modifications [37]. Briefly, bigel samples (5.0 g) were centrifuged in 10 mL tubes at 7104 × *g* for 15 min at 25 °C (model SC-04, Zonki, Foshan, China). The content of Rb1 dissolved in methanol from the separated hydrogel phase was then quantified using an HPLC system (4.6 mm × 250 mm, 5 μm) (model LC-2050, Shimadzu, kyoto, Japan) at 30 °C with a detection wavelength of 203 nm. Mobile phases were acetonitrile (A) and water (B) with a flow rate of 1.0 mL/min, and an injection volume of 10 μL was used to calculate the embedding rate.

### 4.8. Fourier Transform Infrared (FTIR) Spectroscopy

Freeze-dried samples were analyzed using a Fourier transform infrared spectrometer (Shimadzu, Japan) with potassium bromide as the reference matrix. Spectra were acquired over the wavenumber range of 4000–400 cm^−1^ at a 4 cm^−1^ resolution, accumulating 16 scans per measurement. The infrared spectrum of the samples was obtained by subtracting the background spectrum, and a new background spectrum was collected every three scans to eliminate environmental interference. All analyses were performed under constant temperature conditions (25 ± 2 °C).

### 4.9. Differential Scanning Calorimetry (DSC) Analysis

As described by Liu et al. (2020), thermodynamic properties were analyzed using differential scanning calorimetry (Shimadzu, Japan) with a temperature scan from 30 to 80 °C at 10 °C/min under a nitrogen atmosphere [18].

### 4.10. Determination of Rheological Properties

Rheological measurements were performed using a MARS 60 rheometer (Thermo Fisher Scientific, Waltham, Germany) equipped with a 20 mm diameter stainless steel cone-plate geometry set at a constant gap of 0.5 mm. Strain amplitude sweeps (0.01–1000%) were performed at a fixed frequency of 1 Hz.

#### 4.10.1. Stress Scanning

The linear viscoelastic region (LVR) of the bigels was determined by strain scanning. A P35 probe with a diameter of 35 mm was used for testing. The constant shear frequency was set at f = 1 Hz; the gap was 1 mm, and the temperature was maintained at 25 °C. An appropriate amount of the sample was placed on the plate to test the change in the storage modulus (G′) and loss modulus (G″) from 0.1 Pa to 100 Pa.

#### 4.10.2. Flow Curve

As described by Shakouri et al. (2025), a plate with a diameter of 35 mm and a gap of 1 mm was used to determine the flow curve, with a test temperature of 25 °C and a stress set to 1 Pa [38]. The frequency was 1 Hz. An appropriate amount of the sample was placed on the plate, and the change in sample viscosity (η) was measured at shear rates ranging from 0 to 20/s.

#### 4.10.3. Temperature Curve

The same plate with a diameter of 35 mm and a gap of 1 mm was used. An appropriate amount of the bigel sample was placed on the plate with a fixed stress of 1 Pa and a frequency of 1 Hz. With a heating rate of 1 °C/min, the temperature was increased from 20 °C to 80 °C, and the curve of G′ as a function of temperature change was obtained.

#### 4.10.4. Frequency Scanning

According to Li et al. (2020), a strain force of 1 Pa was maintained within the LVR region under a constant low level of strain. The P35 probe was selected along with a 35 mm diameter plate and a gap of 1 mm, with a test temperature of 25 °C [39]. An appropriate amount of the sample was placed on the plate to determine the change curve of G′F and the phase transition angle (δ) in the frequency range of 0.01 to 10 Hz.

### 4.11. In Vitro Digestion Analysis

According to the method by Hashemi et al. (2024) and Kaimal et al. (2023), with slight modifications, 5.0 g of the bigel was added to 20 mL of simulated gastric fluid (SGF), and the pH was adjusted to 3.0 with 1 mol/L HCl, and then the samples were incubated at 37 °C for 2 h. Samples were withdrawn each hour for gastric digestion simulation experiments. Subsequently, 10 mL of the simulated intestinal fluid (SIF) was added to the gastric digestion solution mentioned above, and the pH was adjusted to 7.0 with 1 mol/L NaHCO_3_, and then the samples were incubated at 37 °C for 2 h. Samples were withdrawn each hour for intestinal digestion simulation experiments [40,41]. The bigel system containing Rb1 was subjected to digestion experiments in simulated gastric and intestinal juices. Samples were collected hourly to assess drug release. The percentage of released Rb1 was calculated using the following formula:

$$release\;rate\left(\%\right)=\frac{Rb1released\;during\;digestion}{content\;of\;Rb1in\;digestive\;juice}\times 100$$

### 4.12. Data Processing and Analysis

All data and charts in this experiment were statistically analyzed and plotted using the SPSS 21, Excel 2019, and Origin 2017 software.

## Figures and Tables

**Table 1:** Texture characteristics of different mixing ratios of oleogels and hydrogels.

Oleogel/Hydrogel (*w*/*w*)	Hardness (gf)	Cohesiveness	Chewiness (gf)	Springiness (mm)	Gumminess (gf)
1:0 (−)	928.62 ± 0.64 ^a^	0.27 ± 0.01 ^e^	64.10 ± 0.04 ^a^	0.26 ± 0.00 ^e^	247.03 ± 0.01 ^a^
1:9 (+)	33.25 ± 0.38 ^f^	0.79 ± 0.01 ^ab^	20.53 ± 0.01 ^f^	0.78 ± 0.00 ^c^	26.44 ± 0.06 ^f^
2:8 (+)	43.25 ± 0.33 ^e^	0.75 ± 0.01 ^cd^	25.56 ± 0.01 ^e^	0.77 ± 0.01 ^c^	32.97 ± 0.12 ^e^
3:7 (+)	47.56 ± 0.38 ^d^	0.76 ± 0.01 ^bc^	28.98 ± 0.02 ^d^	0.80 ± 0.00 ^b^	36.38 ± 0.01 ^d^
4:6 (+)	67.96 ± 0.48 ^c^	0.75 ± 0.01 ^cd^	40.44 ± 0.05 ^c^	0.79 ± 0.00 ^b^	51.32 ± 0.01 ^c^
5:5 (+)	79.85 ± 0.38 ^b^	0.81 ± 0.02 ^a^	53.28 ± 0.10 ^b^	0.83 ± 0.00 ^a^	63.95 ± 0.01 ^b^
0:1 (+)	31.74 ± 0.96 ^f^	0.72 ± 0.01 ^d^	17.27 ± 0.01 ^g^	0.76 ± 0.00 ^d^	22.73 ± 0.01 ^g^

Notes: The symbols (+) and (−) represent Rb1, respectively. There are significant differences between different lowercase letters in the same column (*p* < 0.05).

## Data Availability

The original contributions presented in this study are included in the article. Further inquiries can be directed to the corresponding authors.

## Abbreviations

The following abbreviations are used in this manuscript:

Rb1	Notoginsenoside Rb1
WHC	Water-holding capacity
OHC	Oil-holding capacity
EE	Encapsulation efficiency
GI	Gastrointestinal
